# Systemic Immune-Inflammation Index Predicts Contrast-Induced Acute Kidney Injury in Patients Undergoing Coronary Angiography: A Cross-Sectional Study

**DOI:** 10.3389/fmed.2022.841601

**Published:** 2022-03-16

**Authors:** Hangpan Jiang, Duanbin Li, Tian Xu, Zhezhe Chen, Yu Shan, Liding Zhao, Guosheng Fu, Yi Luan, Shudong Xia, Wenbin Zhang

**Affiliations:** ^1^Department of Cardiology, The Fourth Affiliated Hospital, College of Medicine, Zhejiang University, Yiwu, China; ^2^Department of Cardiology, Sir Run Run Shaw Hospital, College of Medicine, Zhejiang University, Hangzhou, China; ^3^Key Laboratory of Cardiovascular Intervention and Regenerative Medicine of Zhejiang Province, Hangzhou, China; ^4^Department of Cardiology, The First Affiliated Hospital, College of Medicine, Zhejiang University, Hangzhou, China

**Keywords:** contrast-induced acute kidney injury, systemic immune-inflammation index, inflammation, coronary angiography, percutaneous coronary intervention, coronary artery disease

## Abstract

**Background and Aims:**

Systemic immune-inflammation index (SII) is an emerging indicator and correlated to the incidence of cardiovascular diseases. This study aimed to explore the association between SII and contrast-induced acute kidney injury (CI-AKI).

**Methods:**

In this retrospective cross-sectional study, 4,381 subjects undergoing coronary angiography (CAG) were included. SII is defined as neutrophil count × platelet count/lymphocyte count. CI-AKI was determined by the elevation of serum creatinine (Scr). Multivariable linear and logistic regression analysis were used to determine the relationship of SII with Scr and CI-AKI, respectively. Receiver operator characteristic (ROC) analysis, structural equation model analysis, and subgroup analysis were also performed.

**Results:**

Overall, 786 (17.9%) patients suffered CI-AKI after the intravascular contrast administration. The subjects were 67.1 ± 10.8 years wold, with a mean SII of 5.72 × 10^11^/L. Multivariable linear regression analysis showed that SII linearly increased with the proportion of Scr elevation (β [95% confidence interval, CI] = 0.315 [0.206 to 0.424], *P* < 0.001). Multivariable logistic regression analysis demonstrated that higher SII was associated with an increased incidence of CI-AKI ([≥12 vs. <3 × 10^11^/L]: odds ratio, OR [95% CI] = 2.914 [2.121 to 4.003], *P* < 0.001). Subgroup analysis showed consistent results. ROC analysis identified a good predictive value of SII on CI-AKI (area under the ROC curve [95% CI]: 0.625 [0.602 to 0.647]). The structural equation model verified a more remarkable direct effect of SII (β = 0.102, *P* < 0.001) on CI-AKI compared to C-reactive protein (β = 0.070, *P* < 0.001).

**Conclusions:**

SII is an independent predictor for CI-AKI in patients undergoing CAG procedures.

## Introduction

Coronary artery disease (CAD) is a very common cardiovascular disease and remains the major cause of mortality worldwide (1). The interventional strategies especially coronary angiography (CAG) and percutaneous coronary intervention (PCI) greatly improve clinical outcomes of CAD patients (2). However, the complications of CAG or PCI pose a significant challenge in clinical practice (3).

Contrast-induced acute kidney injury (CI-AKI) is an iatrogenic acute renal dysfunction resulting from the injection of iodinated contrast agents and becomes the third leading cause of acute kidney injury (AKI) (4). In accordance with European Society of Urogenital Radiology (ESUR), the definition of CI-AKI is that the serum creatinine (Scr) levels elevate more than 44.2 μmol/L (0.5 mg/dl) or 25% from baseline within 72 h after administration of contrast media (5, 6). The incidence of CI-AKI is reportedly high worldwide and varies from 4.4 to 22.1% according to different definitions (7). Various risk factors, including diabetes, heart failure severity, anemia, renal dysfunction and circulatory insufficiency, have been identified to promote the occurrence of CI-AKI (8–10). Of the various factors associated with the CI-AKI, inflammation has been demonstrated to be an important risk factor and inflammation-based predictors can effectively determine high-risk CI-AKI (11, 12).

Many inflammatory markers, including C-reaction protein (CRP), platelet (PLT) and neutrophil-lymphocyte ratio (NLR) have been found to be related to the incidence of CI-AKI (13). However, when only focus on individual parameters, these biomarkers might become unstable and are often impacted by other confounding factors (14). Nowadays, the systemic immune-inflammation index (SII), a simple and comprehensive indicator, has been put forward (15). Based on three inflammatory biomarkers, including PLT, neutrophils and lymphocytes, SII comprehensively reflects the inflammatory and immune status of patients at the same time (16). SII index has been of common use to predict the incidence and evaluate the prognosis of various cancers (17). Recently, researches also demonstrated that SII was associated with the clinical prognosis in CAD patients after coronary intervention (18). However, the association between SII and the CI-AKI risk remains undetermined. Therefore, this retrospective cross-sectional study was conducted to explore the relationship of SII index with CI-AKI in CAD patients who underwent CAG or PCI.

## Materials and Methods

### Study Design and Setting

**Figure 1:**
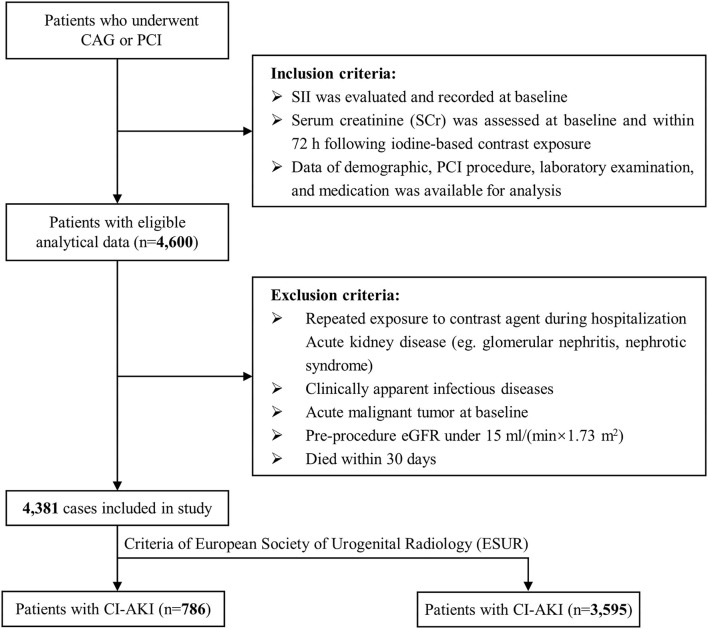
Flowchart of inclusion and exclusion of study population. PCI indicates percutaneous coronary intervention; eGFR, estimated glomerular filtration rate.

The present study utilized a retrospective cross-sectional design and strictly followed the Strengthening the Reporting of Observational Studies in Epidemiology (STROBE) guideline (19). From December 2006 to December 2019, 11,028 consecutive individuals who underwent selective CAG or PCI at Sir Run Run Shaw Hospital and its medical consortium hospitals were eligible for screening. Individuals must meet the following inclusion criteria: (1) patients with available systemic immune-inflammation index (SII) at baseline; (2) patients with recorded Scr at baseline and within 72 h following contrast exposure; (3) patients with complete data of demographics, laboratory data and therapeutic interventions. Exclusion criteria were listed as following: (1) patients suffering repeated exposure of contrast agent during hospitalization; (2) patients with active kidney disease (e.g., glomerular nephritis, nephrotic syndrome); (3) patients with clinically apparent infectious diseases on admission and during hospitalization; (4) patients with malignant tumor at baseline; (5) patients with preprocedure estimated glomerular filtration rate (eGFR) <15 ml/min/1.73 m^2^; (6) patients who died within 30 days after PCI. Finally, 4,381 subjects were included in the present study (Figure 1). The study adhered to the Declaration of Helsinki and received ethical approval from the independent Ethics Committee of Sir Run Run Show Hospital (NO.20201217-36).

### Data Collection and Definitions

All demographic features and clinical data of patients were obtained from the Hospital Information System (HIS), including age, sex, weight status, comorbidities, past medical history, current medications and CAG or PCI procedure-related data.

All the blood biochemical tests were performed by clinical laboratory technicians in hospital certificated laboratory and the results were documented. Peripheral PLT, neutrophil (N), and lymphocyte (L) counts were measured by Automatic Blood Cell Counter (XE-2100, Sysmex, Kobe, Japan). SII was calculated as: SII = PLT × N/L (16).

The baseline Scr concentrations were measured on admission for all patients. The postoperative Scr was measured at least three times within 72 h and the highest recorded value was used for analysis. In accordance with the European Society of Urogenital Radiology (ESUR), CI-AKI is defined as an increase in Scr level ≥ 0.5 mg/dl (44.2 μmol/L) or 25% from baseline within 72 h after administration of contrast agents (5, 6).

### Statistical Analysis

Categorical variables were presented as counts (percentage) and were compared by the Chi-square test or Fisher's exact test. Continuous variables were presented as mean ± standard deviate (SD) and compared by Student *t*-test if distributing normally, while continuous variables were presented as median (interquartile range) and compared by Mann-Whitney *U*-test if distributing non-normally.

The association between preoperative SII scores and the proportion of elevated Scr was validated by multivariable linear regression analysis. The relationship of SII with CI-AKI was determined by multivariable logistic regression analysis and visualized by restricted cubic spline (RCS) curve. Tests for trend (P for tend) were also conducted by treating the ordered SII categories (<3, 3-6, 6-9, 9-12, and ≥12 × 10^11^/L) as a continuous variable in the logistic regression models. The covariates incorporated in multivariate regression models all had great clinical significance of CI-AKI, which were verified by previous studies (20). Receiver operator characteristic (ROC) analysis with Youden index, by using R package “ROCit,” was set to determine the optimal cut-off point of SII, and evaluate the predictive performance of SII on CI-AKI. Structural equation modeling was performed to compare the effects of different inflammatory indicators on CI-AKI. Finally, exploratory analysis was performed among prespecified subgroups.

The two-tailed *P*-value < 0.05 was of statistical significance. All the analysis were conducted with SPSS version 22.0 (SPSS Inc, Chicago, USA), AMOS version 21.0 (SPSS Amos; IBM, Chicago, IL, USA) and R version 4.0.5 (The R Foundation for Statistical Computing, Vienna, Austria).

## Results

### Population Characteristic and Population Distribution

Totally, 4,381 patients undergoing CAG or PCI were eventually included in this study. Table 1 presented the baseline demographics and clinical parameters of enrolled patients. The mean age was 67.1 years old and 66% were male. Among these, 786 subjects suffered from CI-AKI after CAG or PCI. Patients in CI-AKI group had a greater burden of diabetes mellitus (28.2 vs. 23.3%, *P* = 0.004), and tended to receive more beta-blocker, while less statin and aspirin (all *P* < 0.05). Besides, these subjects had aw higher level of CRP and Scr elevation, while a lower level of total cholesterol, high density lipoprotein and triglyceride (all *P* < 0.05). The difference in contrast volume was not significant between groups (80.0 [51.3, 140.0] mg vs. 80.0 [50.0, 130.0] mg, *P* = 0.258). However, CI-AKI patients had a higher level of SII before CAG or PCI procedure (7.66 [4.48, 14.81] × 10^11^/L vs. 5.41 [3.60, 8.75] × 10^11^/L, *P* < 0.001).

**Table 1:** Baseline characteristics of patients according to SII and CI-AKI.

	**Contrast-induced acute kidney injury**			
	**Overall (*n* = 4,381)**	**No (*n* = 3,595)**	**Yes (*n* = 786)**	***P* value**
**Demographic features**				
Age, yrs	67.11 ± 10.76	66.62 ± 10.73	69.34 ± 10.65	<0.001-
Male, *n* (%)	2,892 (66.0)	2419 (67.3)	473 (60.2)	<0.001^*^
BMI, kg/m^2^	24.45 ± 5.33	24.35 ± 5.34	24.96 ± 5.25	0.007^*^
Diabetes, *n* (%)	1,058 (24.1)	836 (23.3)	222 (28.2)	0.004^*^
Hypertension, *n* (%)	2,785 (63.6)	2,272 (63.2)	513 (65.3)	0.293
EF, %	59.72 ± 13.03	60.41 ± 12.87	56.57 ± 13.30	<0.001^*^
Suffered CI-AKI, *n* (%)	786 (17.9)	0 (0.0)	786 (100.0)	<0.001^*^
**Laboratory data**				
SII, ×10^11^/L	5.72 [3.73, 9.51]	5.41 [3.60, 8.75]	7.66 [4.48, 14.81]	<0.001^*^
Scr at baseline, μmol/L	76.0 [64.0, 94.0]	76.0 [65.0, 93.0]	73.0 [60.0, 100.0]	0.082
Scr elevation, %	5.2 [−3.9, 18.2]	1.9 [−5.7, 10.1]	43.1 [32.4, 68.1]	<0.001^*^
NT-proBNP, ng/ml	6.41 [1.58, 2.03]	5.13 [1.29, 16.81]	15.20 [4.80, 36.37]	<0.001^*^
eGFR, ml/(min ×1.73 m^2^)	84.4 [65.7, 94.6]	84.6 [67.1, 94.5]	83.0 [57.2, 95.0]	0.014^*^
CRP, mg/L	2.3 [0.9, 8.0]	2.0 [0.8, 6.6]	4.4 [1.4. 16.1]	<0.001^*^
Hemoglobin, g/dl	12.68 ± 1.99	12.86 ± 1.87	11.99 ± 2.24	<0.001^*^
**CAG/PCI procedure data**				
Volume of contrast agent, mg	80.0 [50.0, 130.0]	80.0 [50.0, 130.0]	80.0 [51.3, 140.0]	0.258
Types of contrast agent, *n* (%)				1.000
Isotonic	1,378 (31.6)	1,130 (31.6)	248 (31.6)	
Hypotonic	3,003 (68.4)	2,465 (68.4)	538 (68.4)	
**Lesions location**, ***n*** **(%)**
LM	137 (11.0)	113 (10.8)	24 (11.5)	0.882
LAD	959 (63.9)	773 (62.5)	186 (70.2)	0.023^*^
LCX	358 (26.4)	298 (26.5)	60 (26.1)	0.971
RCA	493 (36.1)	407 (36.0)	86 (36.8)	0.890
Multivessel lesions, (%)	79 (6.7)	65 (6.6)	14 (6.9)	1.000
Total length of stents, mm	38.0 [25.0, 63.0]	39.0 [25.0, 64.0]	36.0 [24.0, 57.0]	0.101
**Medication**, ***n*** **(%)**				
ACEI	702 (16.0)	564 (15.7)	138 (17.6)	0.215
ARB	1,315 (30.0)	1,116 (31.0)	199 (25.3)	0.002^*^
CCB	1,216 (27.8)	1,003 (27.9)	213 (27.1)	0.682
Beta blocker	2,201 (50.2)	1,780 (49.5)	421 (53.6)	0.044^*^
Statin	3,652 (83.4)	3,050 (84.8)	602 (76.6)	<0.001^*^
Aspirin	3,629 (82.8)	3,059 (85.1)	570 (72.5)	<0.001^*^
Furosemide injection	658 (15.0)	419 (11.7)	239 (30.4)	<0.001^*^
Dopamine	1,231 (28.1)	929 (25.8)	302 (38.4)	<0.001^*^

*Categorical data are presented as n(%) and continuous data are expressed as mean ± standard deviation or median (interquartile range). SII indicates systemic immune-inflammation index; CI-AKI, contrast-induced acute kidney injury; BMI, body mass index; EF, ejection fraction; Scr, serum creatinine; NT-proBNP, N-terminal of the prohormone brain natriuretic peptide; eGFR, estimated glomerular filtration rate; CRP, C-reactive protein; LM, left main coronary artery; LAD, left anterior descending artery; LCX, left circumflex artery; RCA, right coronary artery; ACEI, angiotensin converting enzyme inhibitor; ARB, angiotensin receptor antagonist; CCB, calcium channel blocker*.

* *P < 0.05*.

**Figure 2:**
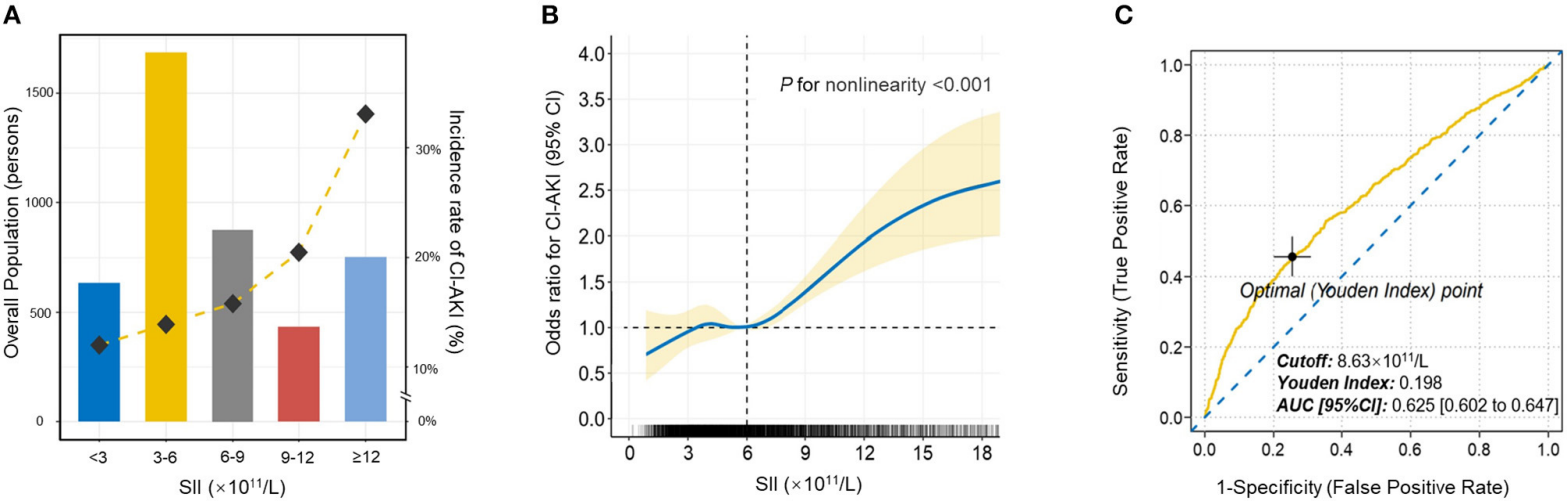
The population distribution histogram, restricted cubic spline analysis and receiver operating characteristic curve. **(A)** The population distribution of the incidence of CI-AKI according to SII. The gold dashed line depicts the changing trend in incidence of CI-AKI. Left axis, population count (persons); right axis, incident rate of CI-AKI (%). **(B)** Restricted cubic spline analysis for exploring the non-linear association between SII and CI-AKI. The solid blue line shows the adjusted odds ratio of SII for CI-AKI, and the shaded area around the solid line indicates 95% confidence interval of the curve. **(C)** Receiver operating characteristic curve of SII for CI-AKI. According to the maximum Youden index, the optimal cut-off value was evaluated and pointed out in the figure. CI-AKI indicates contrast-induced acute kidney injury; SII, systemic immune-inflammation index; AUC indicates area under the curve; CI, confidence interval.

Then, patients were stratified into five distinct categories based on SII scores at equal intervals (predefined cut points: <3, 3-6, 6-9, 9-12, ≥12 × 10^11^/L). The relationship of SII categories with the incidence of CI-AKI was depicted in Figure 2A. As the increase of SII index, the incidence rate of CI-AKI increased correspondingly.

### The Association Between Preoperative SII and the Proportion of Scr Elevation

The univariable and multivariable linear regression analysis were conducted to identify the association between SII and the proportion of Scr elevation (Table 2). The fully adjusted regression model demonstrated that the increased SII scores were closely associated with higher levels of Scr elevation (β = 0.315, 95% CI: [0.206 to 0.424], *P* < 0.001), after adjusting age, sexw, diabetes, hypertension, eGFR, type and volume of contrast agent, EF, CRP, and medications (statin, furosemide injection and dopamine).

**Table 2:** Linear regression analyses of SII on the proportion of elevated Scr.

	**Predictor variable**	**β coefficient [95% CI]**	***P-*value**
Model 1
	SII (×10^11^/L)	0.508[0.402 to 0.615]	<0.001^*^
Model 2
	SII (×10^11^/L)	0.479[0.373 to 0.585]	<0.001^*^
	Age	2.385[1.243 to 3.526]	<0.001^*^
	Male	−3.095[−5.529 to −0.660]	0.013^*^
	Diabetes	2.622[−0.089 to 5.334]	0.058
	Hypertension	−0.977[−3.454 to 1.500]	0.439
	eGFR	0.067[0.009 to 0.124]	0.024^*^
	Type of contrast agent	−1.161[−3.728 to 1.406]	0.375
	Volume of contrast agent	−0.014[−0.030 to 0.002]	0.088
	EF	−0.352[-0.442 to −0.263]	<0.001^*^
Model 3
	SII (×10^11^/L)	0.315[0.206 to 0.424]	<0.001^*^
	Age	2.276[1.158 to 3.395]	<0.001^*^
	Male	−2.528[−4.916 to −0.14]	0.038^*^
	Diabetes	2.442[−0.213 to 5.097]	0.071
	Hypertension	0.453[−1.983 to 2.888]	0.716
	eGFR	0.102[0.045 to 0.159]	<0.001^*^
	Type of contrast agent	−1.581[−4.096 to 0.934]	0.218
	Volume of contrast agent	−0.015[−0.032 to 0.002]	0.081
	EF	−0.121[−0.216 to −0.026]	0.013^*^
	CRP	0.099[0.047 to 0.151]	<0.001^*^
	Statin	−11.318[−14.448 to −8.187]	<0.001^*^
	Furosemide injection	17.592[14.112 to 21.072]	<0.001^*^
	Dopamine	6.479[3.741 to 9.218]	<0.001^*^

*Model 1 adjusted for none*.

*Model 2 adjusted for age (years old), gender (male or female), diabetes (yes or no), hypertension (yes or no), eGFR (ml/min× 1.73 m^2^), type of contrast agent (isotonic or hypotonic), volume of contrast agent (mg), and EF (%)*.

*Model 3 additionally adjusted for CRP (mg/L), and medications (administration of statin, furosemide injection and dopamine) (yes or no)*.

*SII indicates systemic immune-inflammation index; Scr, serum creatinine; eGFR, estimated glomerular filtration rate; EF, ejection fraction; CRP, C-reactive protein*.

* *P < 0.05*.

### The Association Between Preoperative SII and the Occurrence of CI-AKI

Logistic regression analysis was conducted to determine the relationship of SII with the risk of CI-AKI (Table 3). Model 1 demonstrated that higher SII scores were associated with higher risk for CI-AKI when compared to the reference, especially in 6-9, 9-12, and ≥12 × 10^11^/L groups ([6-9 vs. <3]: OR = 1.183, 95% CI [0.897 to 1.560], *p* = 0.037; [9-12 vs. <3]: OR = 1.791, 95% CI [1.247 to 2.572], *P* = 0.002; [≥12 vs. <3]: OR = 2.914, 95% CI [2.121 to 4.003], *P* < 0.001). After further adjusted and fully adjusted, Model 2 and Model 3 also showed similar results (all *P* for trend < 0.001). Furthermore, restricted cubic spline (RCS) model with multiple adjustment was conducted to visualize the relationship between SII and the incidence of CI-AKI (Figure 2B). The results revealed an underlying non-linear relationship that the curve was relatively flat, until SII reached around 6 × 10^11^/L and then began to rise significantly, which was similar to the result of logistic regression (*P* for non-linearity < 0.001).

**Table 3:** Logistic regression analyses of SII on the CI-AKI.

**SII ( ×10^**11**^/L)**	**Cases/overall (%)**	**Model 1**		**Model 2**		**Model 3**	
		**OR [95% CI]**	***P*-value**	**OR [95% CI]**	***P*-value**	**OR [95% CI]**	***P*-value**
<3	76/634 (12.0%)	1 (Reference)		1 (Reference)		1 (Reference)	
3-6	234/1,686 (13.9%)	1.183[0.897 to 1.560]	0.233	1.303[0.977 to 1.738]	0.071	1.228[0.917 to 1.644]	0.168
6-9	138/874 (15.8%)	1.377[1.019 to 1.860]	0.037^*^	1.509[1.100 to 2.070]	0.011^*^	1.332[0.963 to 1.843]	0.083
9-12	89/434 (20.5%)	1.894[1.356 to 2.646]	<0.001^*^	2.165[1.525 to 3.074]	<0.001^*^	1.791[1.247 to 2.572]	0.002^*^
≥12	249/753 (33.1%)	3.627[2.731 to 4.817]	<0.001^*^	3.880[2.872 to 5.241]	<0.001^*^	2.914[2.121 to 4.003]	<0.001^*^
*P* for trend			<0.001^*^		<0.001^*^		<0.001^*^

*Model 1 adjusted for none*.

*Model 2 adjusted for age (per 10 years), gender (male or female), diabetes (yes or no), hypertension (yes or no), eGFR (<30, 30-59, 60-89, or ≥90 ml/min ×1.73 m^2^), type of contrast agent (isotonic or hypotonic), volume of contrast agent (<60, 60-119, or ≥120mg), and EF (<50, 50-64, or ≥65%)*.

*Model 3 additionally adjusted for CRP (<5, 5-10, or ≥10 mg/L), and medications (administration of statin, furosemide injection and dopamine) (yes or no)*.

*CI-AKI indicates contrast-induced acute kidney injury; SII indicates systemic immune-inflammation index; eGFR, estimated glomerular filtration rate; EF, ejection fraction; CRP, C-reactive protein; OR, odds ratio; CI, confidence interval*.

* *P < 0.05*.

### The Assessment of Predictive Ability of SII for CI-AKI

Receiver operating characteristic (ROC) curve was plotted to assess the clinical diagnostic performance of SII on CI-AKI (Figure 2C). ROC curve analysis identified the optimal predictive cut-off value of SII index (cut-off value = 8.63 × 10^11^/L, Youden index = 0.198, AUC [95% CI]: 0.625 [0.602 to 0.647]), which exhibited a satisfactory diagnostic performance.

### The Structural Equation Model Analysis of SII for CI-AKI

**Figure 3:**
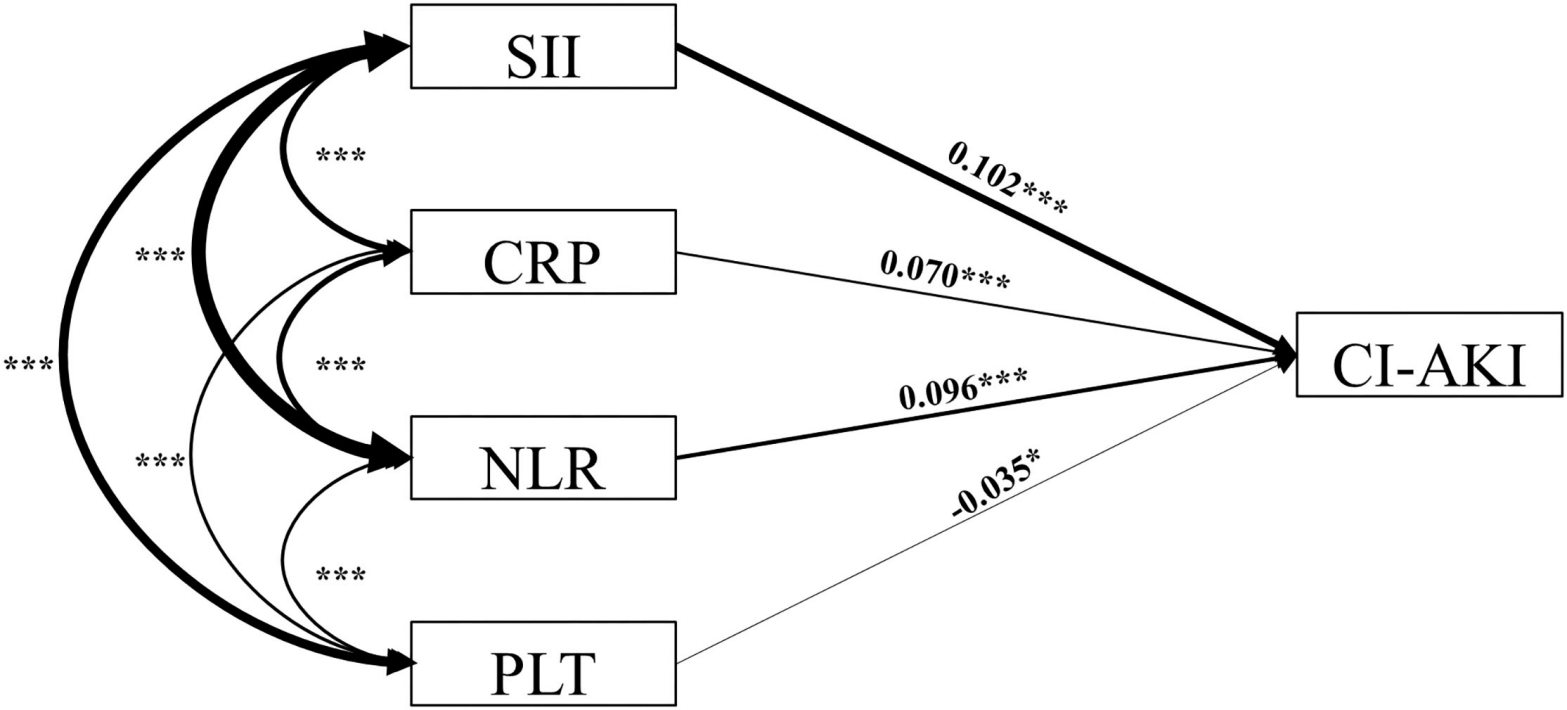
Structural equation model diagram. The structural equation model diagram depicted the relationship between variables by the solid arrows (valid paths). Standardized regression coefficients are presented beside solid arrows. Asterisks indicate the significance levels: (^*^) *P* < 0.05; (^**^) *P* < 0.01; (^***^) *P* < 0.001. The direction (causality) and the degree of correlation of the mutual impacts of the variables are reflected by the direction and thickness of the arrows. SII indicates systemic immune-inflammation index; CI-AKI, contrast-induced acute kidney injury; CRP, C-reaction protein; NLR, neutrophil to lymphocyte ratio; PLT, platelet.

We hypothesized that the direct effect of SII on the incidence of CI-AKI would be stronger when compared with other single inflammatory indicators. To examine the relationship of different inflammatory indicators with CI-AKI, structural equation modeling analysis was conducted (Figure 3; Table 4). The results showed that, compared with NLR (β = 0.096, *P* < 0.001) and PLT (β = −0.035, *P* = 0.030), SII has the most strongly positive association with the incidence of CI-AKI. More importantly, the results also found that the preoperative SII had a closer relationship of CI-AKI than CRP (β = 0.070, *P* < 0.001).

**Table 4:** Results of structural equation modeling analysis.

**Hypothesis**			**Standardized path coefficient**	**Standard error**	***P*-value**	**Support**
SII	→	CI-AKI	0.102	0	<0.001^*^	Yes
CRP	→	CI-AKI	0.07	0	<0.001^*^	Yes
NLR	→	CI-AKI	0.096	0.002	<0.001^*^	Yes
PLT	→	CI-AKI	−0.035	0	0.030^*^	Yes
SII	→	NLR	0.408	89.805	<0.001^*^	Yes
SII	→	PLT	0.371	1149.353	<0.001^*^	Yes
CRP	↔	NLR	0.242	1.828	<0.001^*^	Yes
CRP	↔	PLT	0.108	22.956	<0.001^*^	Yes
NLR	↔	PLT	0.133	5.342	<0.001^*^	Yes

*SII indicates systemic immune-inflammation index; CI-AKI, contrast-induced acute kidney injury; CRP, C-reaction protein; NLR, neutrophil to lymphocyte ratio; PLT, peripheral platelet*.

* *P <0.05*.

### The Exploratory Analysis

**Figure 4:**
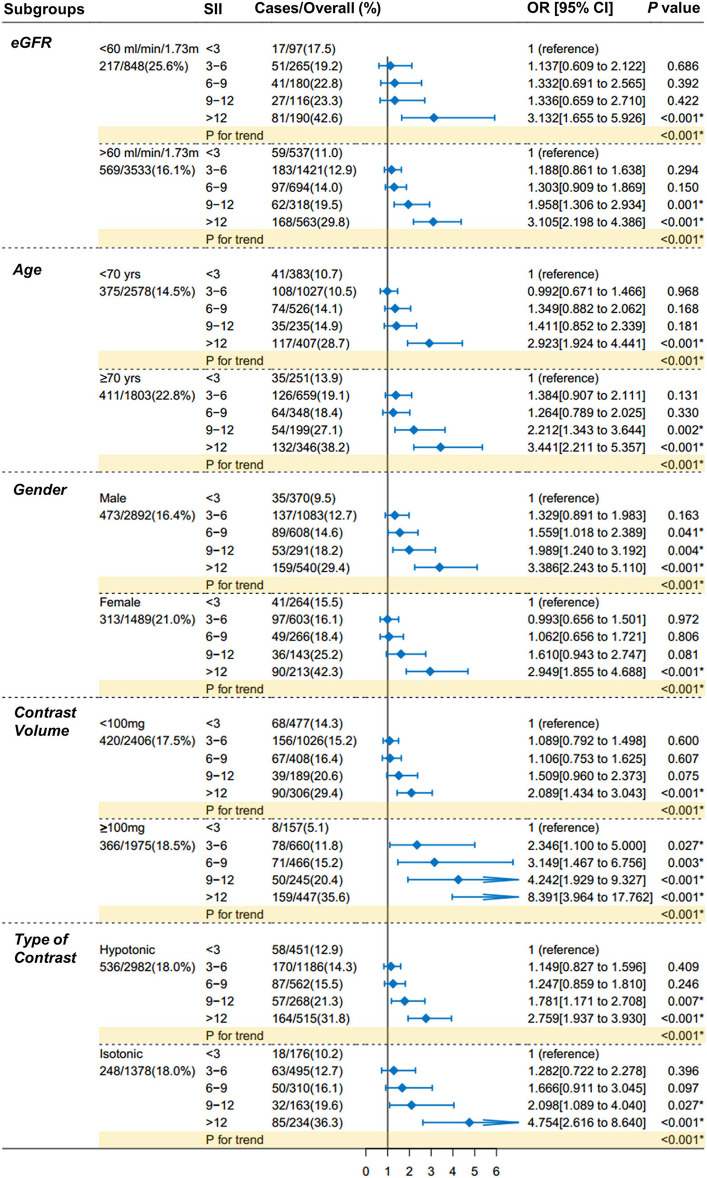
Forest plots of SII for CI-AKI in prespecified subgroups. Patients are dichotomized according to eGFR [ <60 or ≥60 ml/ (min × 1.73 m^2^)], age (<70 or ≥70 yrs.), gender (male or female), exposure volume of contrast agent (<100 or ≥100 mg), and type of contrast agent (hypotonic or isotonic). The increasing trends in the CI-AKI risk with the increase of SII scores were consistent across all subgroups comparing with the main finding (all *P* for trend < 0.001). Multivariable logistic regression in subgroups adjusted the same covariates of Model 3 in Table 3. SII indicates systemic immune-inflammation index; CI-AKI, contrast-induced acute kidney injury; eGFR, estimated glomerular filtration rate.

The exploratory analysis was performed in subgroups (Figure 4), according to eGFR [ <60 or ≥60 ml/(min × 1.73 m^2^)], age (<70 or ≥70 yrs.), gender (male or female), exposure volume of contrast agent (<100 or ≥100 mg), and type of contrast agent (hypotonic or isotonic). The higher risk of CI-AKI with higher levels of SII scores, as compared with the reference group, was consistent across all major subgroups (*P* for trend < 0.001 for all subgroups).

## Discussion

This multicentric retrospective study demonstrated that preoperative elevated SII index was an independent risk factor for the incidence of CI-AKI in CAD patients undergoing CAG or PCI. The preoperative SII index was linearly associated with the elevation of Scr levels. ROC analysis determined the great predictive value of SII for CI-AKI risk. Exploratory analysis also showed similar results in subgroups. More importantly, SII might be the optimal inflammatory indicator to predict the incidence of CI-AKI when not only compared with single NLR and PLT, but also compared with CRP.

Inflammation is well established to contribute to the incidence and progression of multiple diseases (21). Besides, inflammation is also considered as one of the basic mechanisms of CI-AKI, playing a vital role in both initial and subsequent stages of CI-AKI (22). Various inflammatory indicators including CRP, NLR and PLT are found to be closely related to the incidence of CI-AKI (23). Recently, SII, based on neutrophils, lymphocytes and platelets, has been developed as an integrated marker of systemic inflammation (16). Hu et al. firstly reported that SII was a powerful prognostic marker for patients with hepatocellular carcinoma (15). Nowadays, SII has been widely used as a predictive and prognostic indicator in multiple diseases, involving cancers, cardiovascular diseases and kidney diseases (24, 25). Xu et al. reported that SII was associated with acute kidney injury in individuals with hepatocellular carcinoma after hepatectomy (26). Bagci et al. and Kelesoglu et al. also reported that SII was one of the independent predictors of CI-AKI in patients with ST elevation myocardial infarction (STEMI) and non-STEMI, respectively (27, 28). Similar to prior studies, this study also demonstrated that SII was independently associated with CI-AKI risk in patients with CAD and suffering CAG or PCI.

Based on a large-sample dataset, SII seems to be an ideal indicator to identify high-risk CI-AKI patients before CAG/PCI procedure. On the one hand, compared to NLR, SII is a combination of NLR and PLR, which can more comprehensively evaluate the relationship between CI-AKI and inflammation and therefore can provide additional value (29). Studies have demonstrated that SII is a stronger inflammatory marker than NLR or PLR alone with a higher prognostic value (30). On the other hand, structural equation model identified that SII was most strongly associated with the risk of CI-AKI when compared with single NLR, PLT and CRP, which indicated that SII might be a greater indicator to reflect patients' inflammatory status and resulted in increased prediction accuracy of CI-AKI.

The pathogenesis of CI-AKI remains unclear and multifactorial, mainly involving renal vasoconstriction, oxidative stress due to renal medullary hypoxia and direct cytotoxic effects of contrast agents (7, 31). All the factors can result in an inflammatory state (32). As a derived index, high SII score suggests an elevated inflammatory status and decreased immune system. It is of great significance to better understand the combined roles of platelets, neutrophils and lymphocytes to help clarify the association between CI-AKI and SII index. The possible mechanisms between SII and CI-AKI may be speculated as follows. First, inflammation is a prethrombotic state and has been widely recognized to cause thrombosis (33). Not only can endothelial injury state followed by inflammation result in a prothrombotic microvascular environment in kidney vessels, but inflammation cells can downregulate crucial anticoagulant substances to promote microvascular thrombosis (14). Under this inflammatory condition, platelets can be activated by a wide array of inflammatory mediators, such as chemokines, secreted proteins and microRNAs (34). The combined effects of activated coagulant system and downregulated anticoagulant mechanisms therefore cause the elevated platete levels and increase the risk of CI-AKI. Second, experimental researches have reported that the unbalance of inflammatory cells, especially increased neutrophils and decreased lymphocytes, participate in the pathological process of kidney injury (35). The hyperstimulation of neutrophils will lead to the increase of vascular permeability and injured endothelial function (36, 37). Lymphocytopenia results in abnormal immune function, which contribute considerably to the development of renal tissue damage (38). Accordingly, inflammatory surveillance may be beneficial to early identify and prevent the occurrence of CI-AKI during invasive procedures. Third, oxidative stress contributes mainly to the incidence of CI-AKI (39). Enhanced oxidative stress can accentuate the inflammatory cell infiltration and promote an inflammatory state that, in turn, is strictly interconnected with CI-AKI risk (40).

This study also had several limitations that require attention. Firstly, this was a retrospective and observational study and the inherent bias was unavoidable. Therefore, large prospective research should be conducted to support our findings. Secondly, as a multicenter study, there might be differences in detection mode and the abilities of operators, which could lead to bias and error. Thirdly, this study evaluated the inflammatory status of patients upon admission. Medications during hospitalization could affect clinical parameters and haemograms of patients, which might influence the incidence and progression of CI-AKI.

In conclusion, this study demonstrated that in CAD patients who underwent CAG or PCI, preoperative elevated SII index was an independent risk factor of CI-AKI and might be a greater inflammatory indicator in predicting the incidence of CI-AKI than CRP.

## Data Availability Statement

The raw data supporting the conclusions of this article will be made available by the authors, without undue reservation.

## Ethics Statement

The studies involving human participants were reviewed and approved by Ethics Committee of Sir Run Run Show Hospital. Written informed consent for participation was not required for this study in accordance with the national legislation and the institutional requirements.

## Author Contributions

WZ and SX conceived and designed the study. WZ provided the administrative support to this study. HJ organized these data and drafted the manuscript with the help of DL, TX, ZC, YS, and LZ. HJ and DL analyzed the data. ZC drew the pictures. WZ, SX, YL, and GF detected any errors in the whole process. All authors have written and approved the manuscript for submission.

## Funding

This work was supported by grants from the National Natural Science Foundation of China (Nos. 82070408 and 81800212), the Medical Health Science and Technology Project of Zhejiang Provincial Health Commission (No. 2021RC014), and the Traditional Chinese Medicine Science and Technology Project of Zhejiang Province (No. 2021ZB172).

## Conflict of Interest

The authors declare that the research was conducted in the absence of any commercial or financial relationships that could be construed as a potential conflict of interest.

## Publisher's Note

All claims expressed in this article are solely those of the authors and do not necessarily represent those of their affiliated organizations, or those of the publisher, the editors and the reviewers. Any product that may be evaluated in this article, or claim that may be made by its manufacturer, is not guaranteed or endorsed by the publisher.

## Abbreviations

CAD: coronary artery disease
CAG: coronary angiography
PCI: percutaneous coronary intervention
CI-AKI: contrast-induced acute kidney injury
AKI: acute kidney injury
CRP: C-reaction protein
PLT: platelet
NLR: neutrophil to lymphocyte ratio
SII: systemic immune-inflammation index
Scr: serum creatinine
eGFR: estimated glomerular filtration rate
HIS: hospital Information System
PLT: peripheral platelet
ARB: angiotensin receptor antagonists
STEMI: ST elevation myocardial infarction.
